# Superhydrophobic Surfaces Developed by Mimicking Hierarchical Surface Morphology of Lotus Leaf

**DOI:** 10.3390/molecules19044256

**Published:** 2014-04-04

**Authors:** Sanjay S. Latthe, Chiaki Terashima, Kazuya Nakata, Akira Fujishima

**Affiliations:** Photocatalysis International Research Center, Research Institute for Science & Technology, Tokyo University of Science, Noda, Chiba 278-8510, Japan

**Keywords:** lotus leaf, hierarchical, self-cleaning, superhydrophobic, wettability

## Abstract

The lotus plant is recognized as a ‘*King plant*’ among all the natural water repellent plants due to its excellent non-wettability. The superhydrophobic surfaces exhibiting the famous ‘*Lotus Effect*’, along with extremely high water contact angle (>150°) and low sliding angle (<10°), have been broadly investigated and extensively applied on variety of substrates for potential self-cleaning and anti-corrosive applications. Since 1997, especially after the exploration of the surface micro/nanostructure and chemical composition of the lotus leaves by the two German botanists Barthlott and Neinhuis, many kinds of superhydrophobic surfaces mimicking the lotus leaf-like structure have been widely reported in the literature. This review article briefly describes the different wetting properties of the natural superhydrophobic lotus leaves and also provides a comprehensive state-of-the-art discussion on the extensive research carried out in the field of artificial superhydrophobic surfaces which are developed by mimicking the lotus leaf-like dual scale micro/nanostructure. This review article could be beneficial for both novice researchers in this area as well as the scientists who are currently working on non-wettable, superhydrophobic surfaces.

## 1. Introduction

Nature is the world’s giant research laboratory, freely open to all scientists from interdisciplinary fields ranging from Biology, Physics, Chemistry, Mathematics, Materials Science, and Engineering. Nature is fantastic and always ready to teach without hesitation. In Nature, biological micro/nanostructures have developed as the result of millions of years of evolution and their designs sustain many unique and unusual properties [1]. For the past two centuries a majority of the deliberate research and development has been inspired and literally copied from Nature. Numerous next-generation artificial advanced functional materials like nanomaterials and nanodevices have been developed with ease by precisely mimicking the chemical components, surface micro/nanostructures, and exceptional properties present in natural systems [2,3,4]. As the ‘perfect mimicry’ is always impossible, perfect copy of the desired features have not yet been reported. In Mother Nature, numerous plant leaf surfaces exhibit excellent water repellent properties [5,6,7]. Among them, the Sacred Lotus (*Nelumbo nucifera*) is a semi-aquatic plant, which has been popularly known as a symbol of purity in Asian culture for over 2,000 years due to its capability to remain clean. Compared to other natural plant leaves, lotus leaf is the most superhydrophobic, with a water contact angle higher than 160° and sliding angle lower than 5°, hence it always remain clean in muddy and dirty ponds. In the rainy season, when the raindrops fall on the surface of lotus leaves, they immediately bead up like shiny spherical balls and quickly roll off the surface collecting dirt and debris along the way [8]. On the lotus leaf surface, the adhesion between the water droplet and dust particle is stronger than the adhesion between the dusts and the surface, hence the spherical water drops pick up the dust particles while rolling off the lotus leaf. This extreme water repellency and self-cleaning performance of lotus leaf is famously known in the literature as the ‘*Lotus Effect*’ [9]. Furthermore, lotus leaves can retain their superhydrophobicity for a lifetime due to their self-healing function [10]. Before 1996, limited attention was paid to superhydrophobic surface research which was solely based on the relation between static water contact angle and rough surface geometry [11,12,13,14,15]. In 1997, two German botanists, Barthlott and Neinhuis, with the aid of a scanning electron microscope (SEM), revealed for the first time the unique dual scale micro/nanostructure of the lotus leaves and also studied the chemical material present on it [16]. The hierarchical micro/nanostructure of a lotus leaf surface is constituted of secreted low surface energy epicuticular wax crystalloids that uniformly cover the cuticular surface in a regular microrelief of about 1–5 µm in height. It is concluded that the combination of dual scale roughness and low surface energy wax allows air to be trapped under the floating water drops that contribute to the superhydrophobic and self-cleaning behavior of the lotus leaf. This revolutionary research provided two important guidelines for researchers to reactivate the research on superhydrophobic surfaces, one is roughening the low surface energy materials [17,18,19,20], and other is the modification of rough structures with low surface energy materials [21,22,23,24,25]. Thus the unusual surface wettability existing in Nature can be directly mimicked by controlling the surface geometrical microstructure and low surface energy of the surface.

Since this discovery by Barthlott and Neinhuis, plenty of research articles [17,18,19,20,21,22,23,24,25,26,27,28,29,30] and reviews [31,32,33,34,35,36,37,38,39,40] have appeared on the superhydrophobic surfaces describing their adoption in various potential applications ranging from self-cleaning coatings for windshields of automobiles [41], optical devices [42], window glasses and solar panels [43,44], anti-fogging and anti-corrosive coatings [27], paints [45], hydrodynamic drag reduction [46,47], anti-icing [48] and interior fabrics [49]. In many review articles as well as in the research articles, the famous ‘*Lotus Effect*’ has been discussed and the lotus leaf-like micro/nanoscale binary structure is recommended for the development of superhydrophobic surfaces [2,3,6,7,8,36,38,41]. Here we made an attempt to provide a review article which describes in particular the state-of-the-art research into the synthesis of artificial superhydrophobic surfaces by mimicking the lotus leaf-like micro/nanostructure. We thoroughly summarize the different design and fabrication strategies as well as their potential applications in non-wetting surfaces. We also intended to bring in light the different wetting properties of lotus leaves other than just high water contact angle and low sliding angle. This review article is organized into five main sections. The first section presents a brief introduction to a natural superhydrophobic surface, lotus leaves, including the dependence of their surface wetting properties on the chemical composition and rough hierarchical surface morphology. In second section, the different wetting properties of a solid surface by a liquid are briefly introduced. The third section provides a comprehensive overview on the different wetting properties of lotus leaves studied other than high water contact angle and low sliding angle. The fourth section is again organized in two subsections, in first subsection, the superhydrophobic surfaces prepared by using lotus leaves as a biological template are discussed, while in other subsection different physical and chemical methods used for the preparation of lotus leaf-like micro/nanostructures are presented. Finally, we will present our perspective on the future directions and challenges in the fascinating research area of superhydrophobic surfaces developed by mimicking the lotus leaf-like surface morphology.

## 2. Wetting Properties of a Solid Surface

**Figure 1:**
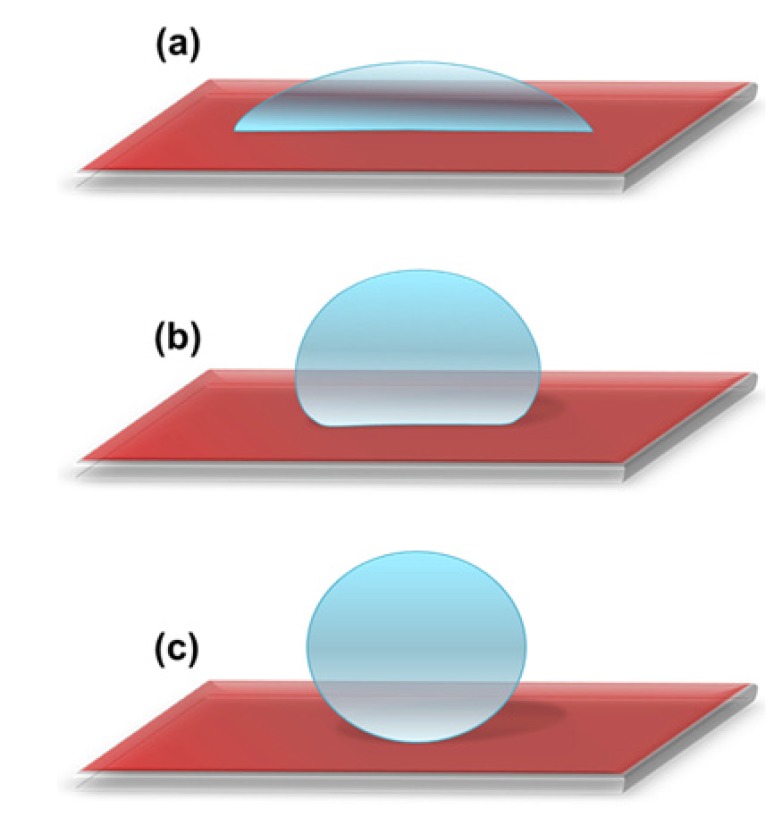
A schematic showing (**a**) hydrophilic surface with water contact angle less than 90°; (**b**) hydrophobic surface with water contact angle greater than 90° and (**c**) superhydrophobic surface with water contact angle larger than 150°.

Based on how the water drop interacts with a solid surface, the surface can be categorized as hydrophilic, hydrophobic or superhydrophobic. Water contact angle measurements are often used to characterize the wettability of the solid surface. A hydrophilic surface shows strong affinity towards water, whereas a hydrophobic surface strongly repels water [50]. The surface is known to be hydrophilic when the water contact angle is less than 90° (Figure 1a), hydrophobic when the water contact angle is greater than 90° (Figure 1b) and superhydrophobic when the water contact angle is larger than 150° (Figure 1c).

**Figure 2:**
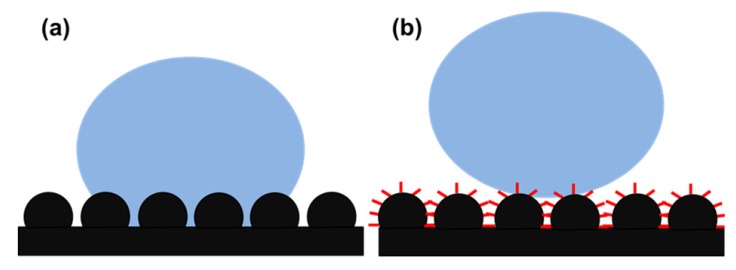
A schematic showing the behavior a water droplet on a rough surface in (**a**) Wenzel’s and (**b**) Cassie-Baxter’s state.

To understand the correlation between the surface roughness and wettability, the famous Wenzel and Cassie-Baxter models have been proposed. The behavior a water droplet on a rough surface in both Wenzel’s [11] and Cassie-Baxter’s [12] state are schematically shown in Figure 2. A water drop can either fill the rough structure or sit above the rough structure. In Wenzel’s model, the liquid completely fills the rough structure of the solid surface after contact (Figure 2a). Wenzel proposed that with increase in surface roughness, a hydrophobic surface will become more hydrophobic, whereas a hydrophilic surface will become more hydrophilic. When dealing with a dual scale surface structure, the Wenzel model is not satisfactory and therefore the Cassie-Baxter model was proposed [51]. In the case of the Cassie-Baxter model, the liquid drop sits on the top asperities of dual scale surface structure and air is supposed to be trapped in rough structure underneath the liquid, which gives a high water contact angle (Figure 2b).

## 3. Different Wetting Properties of Lotus Leaf

**Figure 3:**
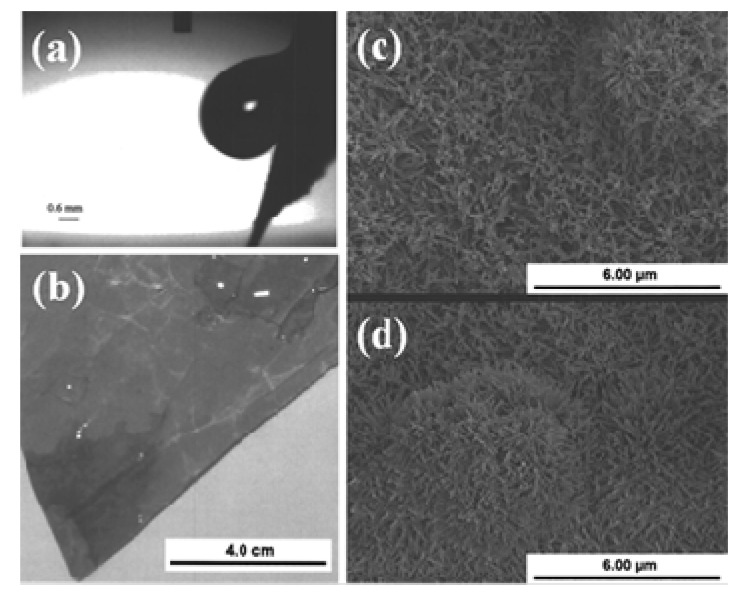
(**a**) A water drop placed on a water condensed lotus leaf and then tilted to 73.7°, (**b**) water condensed lotus leaf with hydrophilic regions, and SEM images of (**c**) a water condensed lotus leaf’s hydrophilic region and (**d**) an untreated lotus leaf. Images reprinted from [53], with permission from AIP Publishing LLC, Copyright 2005.

Apart from high water contact angles and low sliding angles, the lotus leaf exhibits different wetting behaviors under diverse conditions. After the first attempt by Barthlott and Neinhuis, many researchers have studied the micro/nanostructure and different wetting properties of lotus leaf under diverse conditions. Recently, Ensikat *et al.* [52] systematically compared the relevant morphological, chemical, and mechanical features of lotus leaves with the other superhydrophobic plant leaves in order to show their significance. It is well recognized that water drops quickly roll off lotus leaf surfaces at small tilt angles; however this is not the case when water is condensed on the surface of leaves. Cheng *et al.* in their letter [53] raised an interesting fundamental question: “*Is the lotus leaf truly superhydrophobic?*” and concluded that the lotus leaves can be either hydrophobic or hydrophilic, depending on how the water interacts with leaf surface. They performed water condensation experiment on lotus leaves, in which most of the leaf was covered with different sized condensed water droplets without rolling off. After dropping water drops on this water condensed lotus leaf, in some regions, the droplet hit the surface and rolled off due to high contact angle, collecting small water droplets along the way, however in other areas, water drops hit and stopped on the surface exhibiting high contact angle and remained adhered, even after tilting the leaf close to 90° (Figure 3a). In some other areas, water droplets hit the surface and spread out, wetting the leaf with water contact angles of less than 90°, showing hydrophilic behavior (Figure 3b). These water condensed lotus leaves were dried and the water contact angles and tilt angles were measured, which showed recovery of superhydrophobicity. The dual scaled microstructure of the lotus leaf was remained unchanged by the water condensation experiment (Figure 3c,d).

**Figure 4:**
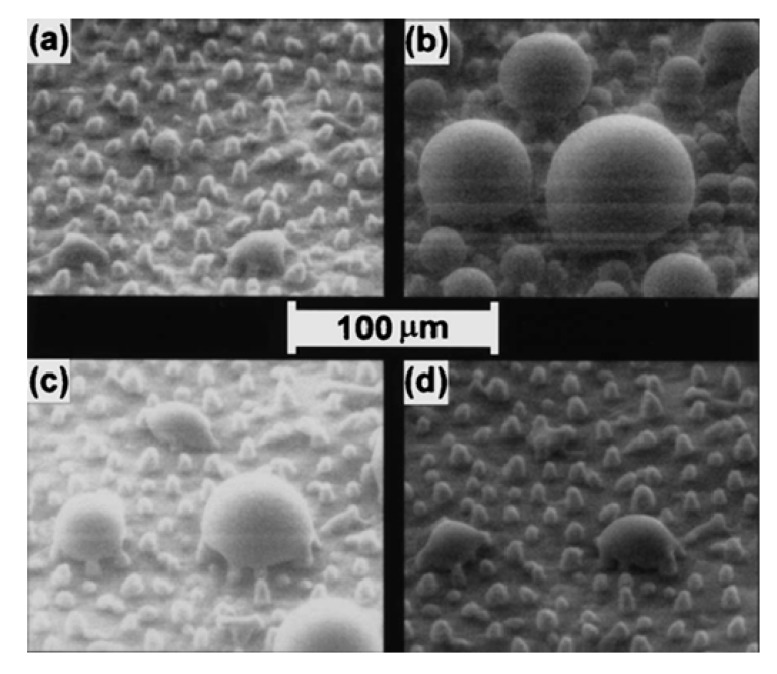
ESEM images captured during water condensation when droplets grow (**a**,**b**), and that during water evaporation when droplets shrink (**c**,**d**) on a lotus leaf. Images reprinted from [54], with permission from AIP Publishing LLC, Copyright 2005.

Thus the Lotus Effect vanishes temporarily when the microscopic water molecules enter the rough hierarchical structure of the lotus leaf during water condensation. This finding can limit the use of superhydrophobic coatings in anti-fogging applications. In continuation with this research, Cheng *et al.* [54] carried out real-time *in situ* microscopic observations of water condensation and evaporation on the surface of lotus leaves using an environmental scanning electron microscope (ESEM). The experimental observations showed that the water drops grow to very high apparent contact angles during water condensation (Figure 4a,b) but they show sticky behavior with no roll-off even when the leaf is tilted by 45°. This stickiness is due to the fact that water vapors can permeate the hair-like nanostructure of the lotus leaf and make the wetted hair-like regions more hydrophilic, causing strong adhesion between the micro-sized papillae’s and the water drops. The strong adhesion is also evident as droplets shrink in size resulting in a decrease in the contact angle when water evaporates (Figure 4c,d).

**Figure 5:**
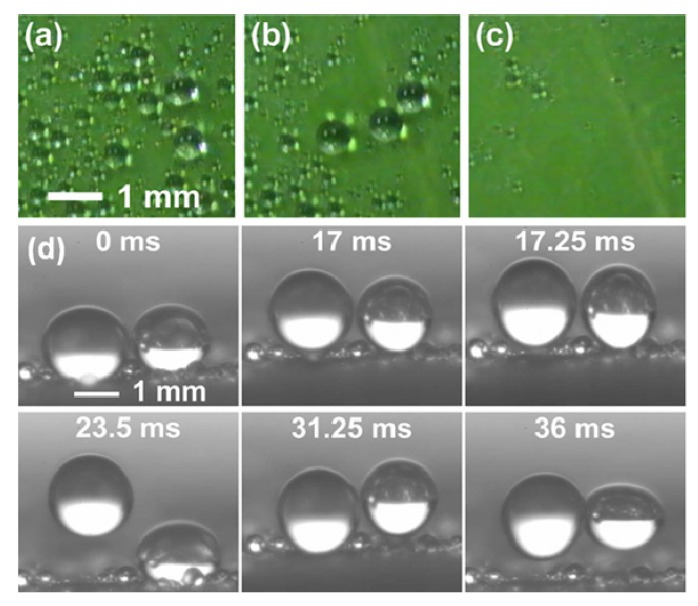
(**a**) Condensed water drops on a horizontally placed lotus leaf surface; (**b**) remaining sticky condensate after the leaf was vertically tilted and returned to the horizontal position; (**c**) the same lotus leaf after vibration; (**d**) millimeter size condensed water drops exhibiting Wenzel to Cassie transition, during vibration at an amplitude of 1 mm and 80 Hz. Images reprinted from [59], with permission from APS, Copyright 2009.

The authors suggested that in the case when the size of water drops and the surface roughness are of the same magnitude, the well-known Wenzel and Cassie-Baxter equations for wetting on rough surfaces may not be applicable. This research also partially answers the question: “*Why do the water drops maintain high contact angles on sticky superhydrophobic surfaces?*” These findings provide significant ramifications on how to develop, employ, and model superhydrophobic surfaces. Recently, the dynamics of contact line depinning [55] and evaporation modes and kinetics of evaporating sessile water droplets [56] on structured superhydrophobic surfaces were investigated experimentally. In other research articles [57,58,59,60], similar water condensation experiments on lotus leaves, and the subsequent study of static and dynamic water contact angles on water-condensed lotus leaves have been reported. It is confirmed from earlier studies that during water condensation, water drops grow and achieve high contact angles on lotus leaf surfaces without rolling off, acquiring a sticky Wenzel state. Boreyko *et al.* [59] proposed that the energy barrier for transition from the sticky Wenzel state to the non-sticky Cassie state can be overcome by using mechanical vibration. In their experiments, water drops of different sizes were condensed on the surface of a lotus leaf (Figure 5a), and the leaf surface was tilted vertically and returned back to horizontal position, whereupon it still showed some sticky water drops on the leaf surface (Figure 5b), then the horizontal lotus leaf was vibrated at 80 Hz and 1 mm peak-to-peak amplitude for 1 s, and all of the sticky drops rolled off except for very small diameter water drops (Figure 5c). The vibration-induced removal of condensed sticky water drops was further clarified by video imaging as depicted in Figure 5d. The Wenzel to Cassie transition of the millimeter size sticky drops into the non-sticky drops was triggered by mechanical vibration, whereas the condensed drops with very small diameters stayed in the sticky Wenzel state indefinitely unless absorbed by larger ones. Such studies are yet not reported in the case of artificially prepared sticky superhydrophobic surfaces.

A substantial change or damage in the microstructure and/or surface chemical composition of the lotus leaf during the water condensation and evaporation experiment can result in loss of its superhydrophobic behavior. Recently, Liu and Choi [60] carefully carried out water condensation and evaporation on lotus leaves without damaging their microstructure and surface chemistry. In their experiments, water condensation was done by exposing pre-cooled lotus leaves (maintained at 4 °C in a refrigerator) to moisture (80% ± 2% RH) at room temperature (23 °C) for 1–10 min, followed by natural evaporation. The wetting state of the lotus leaf during water condensation and evaporation was characterized by evaluating the water contact angle, contact angel hysteresis and surface morphology. They found a mixed wetting state on lotus leaves, a combined classical Cassie-Baxter and Wenzel state that causes a distinct increase of contact angle hysteresis, however, the original Cassie-Baxter state was recovered by applying ultrasonic vibration. Conversely, when the surface is fully wetted (classical Wenzel state), such recovery is not possible with ultrasonic vibration.

**Figure 6:**
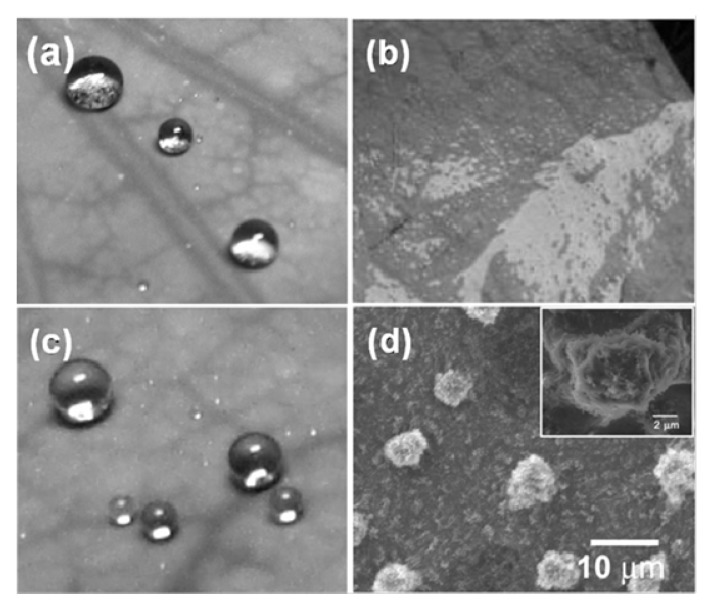
Photographs of lotus surfaces: (**a**) untreated; (**b**) immersed in water at the depth of 50 cm for 2 h; (**c**) dried by nitrogen gas after being immersed; (**d**) ESEM images of microstructures. Images reprinted from [61], with permission from the American Chemical Society, Copyright 2008.

Lei Jiang’s group [61] successfully transformed the highly non-wetting ability of lotus leaves into a complete wetting state (contact angle ~0°) by immersing the leaves in water at a depth of 50 cm for 2 h (Figure 6). The primary and secondary structures with micro/nano arrays of papillae increases the superhydrophobicity of the lotus leaves, as no water invaded between papillae, but after applying the hydraulic pressure to the lotus leaves, the water penetrated the papillae structures which significantly reduces the superhydrophobicity of lotus leaves and they become completely hydrophilic. However the non-wetting behavior of lotus leaves was mostly restored after drying by nitrogen gas. Hence, the dewetting state of lotus leaves depends on the applied hydraulic pressure.

In the same way, Sheng and Zhang [62] also studied the durability of the air layer formed around the lotus leaf underwater by applying a hydraulic pressure (~13.5 kPa). Thus, when developing microdevices floating on or lying under water, the hydraulic pressure should be considered as an important parameter. This is one of the new metrics to check the durability of the superhydrophobic coating in deep water. In some studies, plastron layer formation (a thin air ﬁlm between the solid and liquid interface) after immersing the superhydrophobic surface in water at low immersion depth has been reported [63,64,65,66,67]. These reports suggested that the decay of the plastron layer varies with the type of surface morphology and the surface energy of the superhydrophobic surface. This plastron layer disappears with respect to time and the liquid wets the surface partially or completely. As the nano-scale hairs present on the rough structure of papillae is responsible for the roll off behavior of the water drops on lotus leaves, Cheng *et al.* [68] deliberately removed the nano-scale hair-like structures by thermal annealing (~150 °C for 1 h), keeping the micro-scale roughness and chemical composition almost intact. This change modifies the roughness of lotus leaves resulting in a decrease in contact angle (~126 ± 6°) and the water drops no longer roll off the leaf even after when turned upside down. The loss of nano-scale hair-like features is solely responsible for the transition from weak to strong water adhesion on the lotus leaves. Liu *et al.* [69] studied the stability of the superhydrophobic properties of lotus leaves against hot water. In their controlled experiments, lotus leaves showed a steady decrease in water contact angle from 152° to 120°, when the temperature of water was increased from 25 to 45 °C. However, for water temperatures above 50 °C, the water contact angles decreased well below 40°, confirming the hydrophilic behavior of lotus leaves towards hot liquids. One of the reasons for this is due to a decrease in the surface tension of water when the temperature increases and this low surface tension water easily enters the rough micro/nanostructure of the lotus leaf. However, this behavior is mainly attributed to the damage of the surface micro/nanostructure of the lotus leaves by hot water. It was confirmed by the microstructural analysis that, when the water temperature was lower than the melting point of wax (about 40–50 °C), the hierarchical rough micro/nanostructure of the lotus leaf was stable, however, when the temperature of the water is higher than the melting point of wax, the hierarchical rough structure was affected. The authors also checked the effect of hot water on the wetting properties of artificial superhydrophobic surfaces (CNTs-Teflon treated fabrics), which showed almost identical results as lotus leaves. On this note, if the superhydrophobic surface could repel hot liquids, then such surfaces could be used for next-generation self-cleaning surfaces.

Until now, the self-cleaning properties of the upper side of lotus leaves have been intensively studied, whereas investigations on the wettability of the lower side of a lotus leaf are usually ignored. Recently, Cheng *et al.* [70] discovered an interesting underwater superoleophobic property of the lower side of a lotus leaf (*i*.*e*., Janus Effect), illustrating biological multi-functionality of lotus leaves. The lower side of a lotus leaf shows stable underwater superoleophobicity for apolar and amphiphilic oils as well. The lower side of lotus leaf consists of numerous tabular and slightly convex papillae of 30–50 µm length and 10–30 µm width, which result in underwater superoleophobicity. This Janus feature of the lotus leaf works collectively and keeps the floating lotus leaf clean and tidy in murky water. This appealing study provides an intelligent approach to design and develop bionic multi-functional interface materials. In conclusion, the superhydrophobicity of the lotus leaf cannot be maintained under different vigorous environmental conditions. However, in most of the cases, the superhydrophobicity of the lotus leaf was recovered unless there are some severe damage to the surface structure and surface chemistry.

## 4. Development of Lotus Leaf-Like Surface Morphology to Achieve Superhydrophobicity

### 4.1. Development of Lotus Leaf-Like Surface Structure Using Lotus Leaf Itself as a Template

Templation is an easy and versatile method which has grabbed much attention in biomimetic research, especially to mimic surface morphologies [71]. In the typical templation method, a template with desired properties has been used to prepare a negative replica by molding and this replica was pressed on a soft polymer surface to copy the desired features, particularly the morphological characteristics [72]. This method is alternatively known as a soft lithographic imprinting method which needs neither sophisticated instruments nor complex chemical reactions. The templation method can be applicable to transfer a desired morphology to a range of polymer surfaces at a large scale via roller-printing. The lotus leaf has been used as a biological template to mimic its surface micro/nanostructure on polymer surfaces to achieve similar superhydrophobic wetting properties. In this section, we focus only on the development of lotus leaf-like micro/nanostructure using the lotus leaf itself as a template and hierarchical superhydrophobic surfaces prepared using other templates are not discussed.

**Figure 7:**
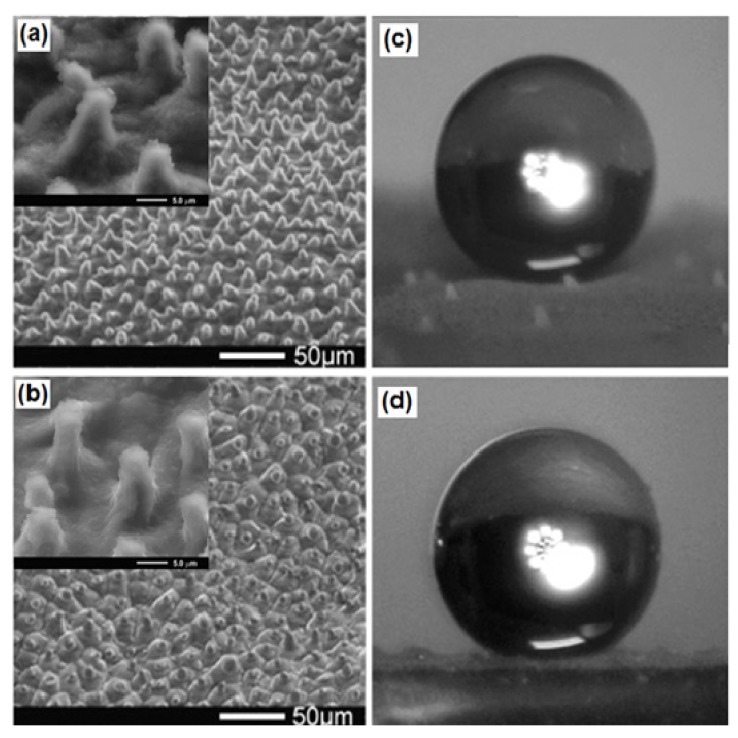
SEM images of: (**a**) the natural lotus leaf and (**b**) the superhydrophobic PDMS surface (respective insets shows the higher magnification images) and spherical water droplets on the (**c**) lotus leaf and (**d**) superhydrophobic PDMS surface. Images reprinted from [73], with permission from American Chemical Society, Copyright 2005.

Since the last decade, many reports on the development of lotus leaf-like surface morphology using the lotus leaf itself as a biological template are available [73,74,75,76,77,78,79,80,81,82,83,84]. Seven years after Barthlott and Neinhuis’s investigation on the surface micro/nanostructure of the lotus leaf, Ji’s research group reported for the first time the development of a superhydrophobic artificial lotus leaf surface using the original lotus leaf as a natural template [73]. Liquid poly(dimethylsiloxane) (PDMS) was cast on fresh lotus leaves and peeled off after solidification, resulting in a negative template. An antistick trimethylchlorosilane (TMCS) monolayer was evaporated on this negative template and again a second PDMS replication was performed on it. In this fashion, the hierarchical surface morphology of the lotus leaf was directly transferred with high precision to the PDMS surface which exhibited similar wetting behavior as the original lotus leaf (Figure 7). However the molding of fresh lotus leaves may create artifacts due to water evaporation from the structure and consequently shrinkage of papillae structures. The same year, Furstner’s group in association with Barthlott and Neinhuis reported the preparation of polyether (PE) replicates of water repellent plant leaves including lotus leaves [78]. Although, it could not possible to replicate nanoscale wax crystalloids, the lotus leaf replica exhibited the highest water contact angle (~157.8°) among all the replicates, which confirms that the morphology formed by the papillae alone could achieve high water repellency. After this research, to date yearly publications on the development of superhydrophobic surface on diverse soft polymer materials using different biological templates have been reported [79,80,81,82,83,84,85,86,87,88].

**Figure 8:**
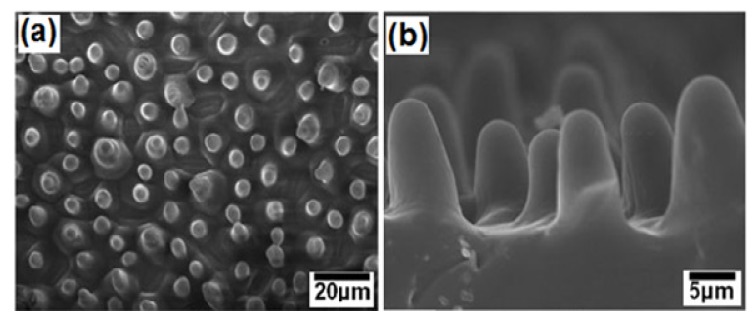
SEM images of the imprinted layers of epoxy-based azo polymer (**a**) a top-view and (**b**) a cross-section view of the surface. Images reprinted from [80], with permission from WILEY-VCH Verlag GmbH & Co. KGaA, Weinheim, Copyright 2006.

Lee and Kwon [79] used nickel molds to prepare two types of superhydrophobic polymeric lotus leaf replicas from intrinsically hydrophobic PDMS by polymer casting (PC) and from intrinsically hydrophilic UV-curable photopolymer by UV-nanoimprint lithography (UV-NIL). The replica produced from PC showed lotus leaf-like morphology with the absence of nano-scaled structure, however it exhibited surface wettability similar to that of a lotus leaf. In contrast, the replica produced from UV-NIL showed nano-scale as well as micro-scale structures identical to those of the lotus leaf, but the sliding angle was higher, even though the contact angle was in a superhydrophobic state. Hence the authors suggested that, to produce superhydrophobic surfaces using a templation method, one should use an intrinsically hydrophobic polymer instead of an intrinsically hydrophilic one. Liu *et al.* [80] inked the substrate with the epoxy-based azo polymer solution and a PDMS stamp having a negative lotus leaf surface structure was pressed on the substrate. After removing the stamp, they obtained nanostructure-free microstructured lotus leaf-like morphology on the substrate (Figure 8) which showed strong superhydrophobicity. Similar to this, using the lotus leaf as a natural biological template, many superhydrophobic surfaces on various polymers like poly(vinyl chloride) (PVC) [81], poly(dimethylsiloxane) (PDMS) [82,83], poly(ethylene oxide) (PEO) [84] have been reported to date.

Even though the template method is extensively used to duplicate the surface texture, it is also not sensitive enough to precisely replicate the epicuticular wax crystalloids present on the lotus leaf surface because they are extremely delicate and small (about 200 nm in diameter). To address this problem, Koch *et al.* [89] removed the epicuticular waxes of the lotus leaves before replication and consequently the replicas were covered with the wax tubule nanostructures of original lotus wax by self-assembly to create hierarchical structures similar to the natural lotus leaf. The hierarchically structured lotus leaf replicas showed improved wetting properties having a static contact angle of 171°, contact angle hysteresis (2°) and tilt angles of 1–2° as compared to natural lotus leaves. This might be due to the wax tubule lengths, which are 0.5 to 1 µm longer in the artificial lotus leaf. The hierarchical structure is responsible for air trapping, resulting in less surface contact area with water droplets and the small contact angle hysteresis and adhesion forces.

### 4.2. Superhydrophobic Surfaces Developed by Mimicking Lotus-Leaf Morphology

The hierarchical surface micro/nanostructure in combination with low surface energy of the lotus leaf is a perfect model for the development of artificial biomimetic superhydrophobic surfaces. Nowadays it has been well accepted that surfaces having rough micro/nanostructures with low surface energy can exhibit water contact angles of nearly 180° and water drops roll off these surface effortlessly. Here we review only those superhydrophobic surfaces which have revealed lotus leaf-like surface morphology and wettability, whereas other rough hierarchical superhydrophobic surfaces were not taken into consideration. To date, there is an enormous amount of reports available on the development of superhydrophobic surfaces which mimic lotus leaf-like surface morphology and low surface energy [90,91,92,93,94,95,96,97,98,99,100,101,102,103,104,105,106,107].

**Figure 9:**
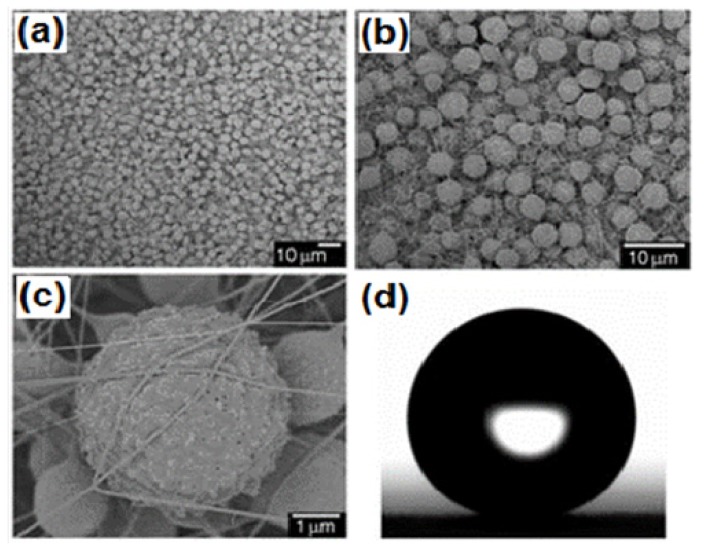
(**a**) SEM image of the film prepared from a 7 wt.% PS/DMF solution; (**b**) 3D network structure of the film; (**c**) surface nanostructure of a single porous microsphere; (**d**) water droplet on the film. Images reprinted from [108], with permission from WILEY-VCH Verlag GmbH & Co. KGaA, Weinheim, Copyright 2004.

In the first decade of the 21st century, Jiang’s group used an electrohydrodynamics (EHD) method to develop a polystyrene (PS) composite film exhibiting lotus leaf-like porous microspheres and nanofibers structure which showed superhydrophobicity without any post chemical modification [108]. The surface micro/nanostructure of the surfaces prepared using EHD can be easily controlled by simply varying the solution concentration. A film prepared from 7 wt% PS/DMF solution showed numerous microspheres (size ~3–7 µm) interlinked with nanofibers (size ~60–140 nm) and the nanofibers are interwoven in a stable multilayer 3D network in which the microspheres are surrounded (Figure 9a,b). The surface of the microspheres contains many bumps and cavities with dimensions of 10–100 nm (Figure 9c). These porous microspheres surrounded by nanofibers contribute to the superhydrophobicity by increasing the surface roughness. A water drop acquires a water contact angle of ~160° on this surface (Figure 9d).

**Figure 10:**
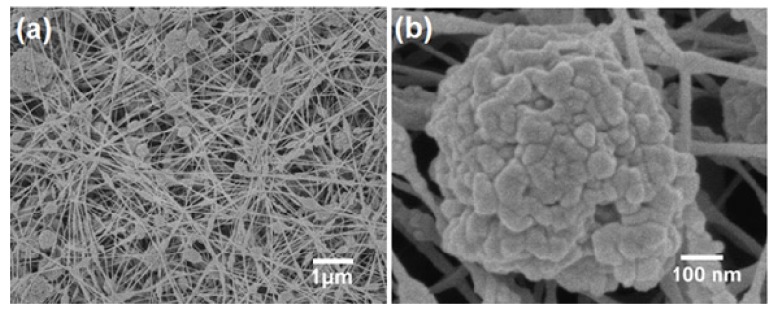
(**a**) SEM image of an electrospun PANI/PS composite film with lotus-leaf-like structure prepared from a 3.72 wt.% PS:ABSA/DMF solution; (**b**) Magnified view of a single sub-microsphere from (**a**). Images reprinted from [109], with permission from WILEY-VCH Verlag GmbH & Co. KGaA, Weinheim, Copyright 2006.

In another study, Jiang’s group reported the preparation of self-cleaning superhydrophobic polyaniline/polystyrene composite films which showed the similar lotus leaf-like micro/nanostructure by a simple electrospinning method [109]. The prepared film exhibited a network of nanofibers with many sub-micrometer-sized spheres distributed on the substrate (Figure 10a). A higher magnification image (Figure 10b) shows that the rough micro/nanostructure is composed of many ‘nanoknots’ connecting the nanofibers together, as well as many nanoscale bumps observed on each sub-microsphere. This self-cleaning superhydrophobic film also showed electrical conductivity due to the use of conducting polyaniline. The wetting properties as well as conductivity of the films were stable in many acidic and basic corrosive solutions over a wide pH range and also in oxidizing solutions. Such multifunctional films can find potential industrial applications in anticorrosive coatings, electromagnetic-interference shielding, and antistatic coatings.

**Figure 11:**
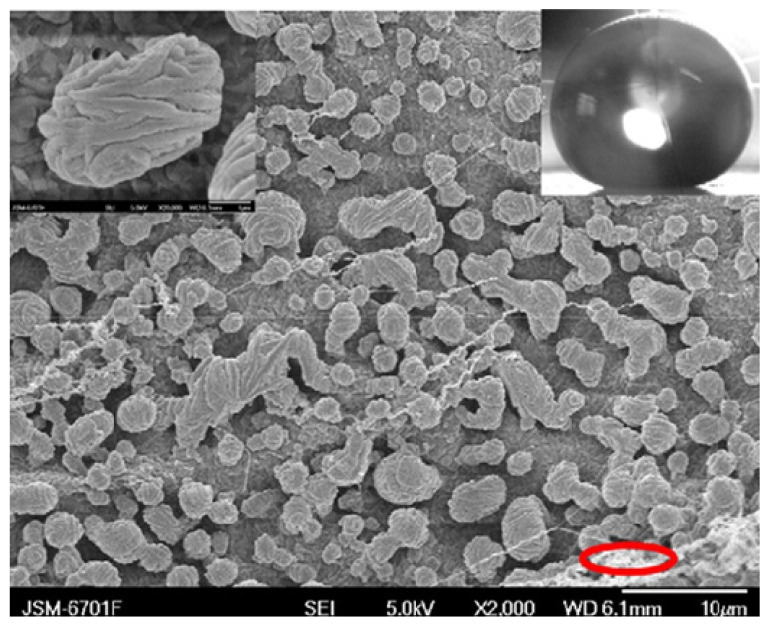
SEM image of the as-prepared PTFE surface sintered at 360 °C (left inset shows single papillae with rough morphology and right inset shows the spherical water drop on the surface with contact angle of 162 ± 2°). Images reprinted from [111], with permission from Elsevier, Copyright 2009.

Mao *et al.* [110] prepared polystyrene (PS) nanotube films using a melting method which exhibited surface micro-nanostructures similar to the micropapillae on a lotus leaf. Each PS micropapilla was assembled from numerous 200 nm diameter PS nanotubes (identical to the hair-like nanostructures on the micropapillae of a lotus leaf). For the preparation of hierarchical PS nanotube films, blank PS film was lightly pressed with a porous alumina membrane template for 12 h. After 12 h, the template was dissolved in NaOH solution to afford the PS nanotube film. This structure exhibited strong superhydrophobicity and good blood compatibility. The blood compatibility of PS nanotube film was achieved by just controlling the geometrical shape of the surface. Hou and Wang [111], used filter paper for the first time as a template to prepare large area superhydrophobic polytetrafluoroethylene (PTFE) surfaces exhibiting lotus leaf-like microstructures. A filter paper can be decomposed at high temperature and the template is removed during the sintering process, leaving the rough superhydrophobic PTFE surface behind. The PTFE surface sintered at a temperature of 360°C showed lotus leaf-like microstructures, having many rough papillae with a diameter about 3–5 µm (Figure 11). This surface showed superhydrophobic behavior with a water contact angle of 162 ± 2° and a sliding angle of 3°. The number of papillae on the surface was found to decrease with increasing sintering temperature. The superhydrophobic PTFE surface showed excellent stability against acid, alkali or organic solvents.

**Figure 12:**
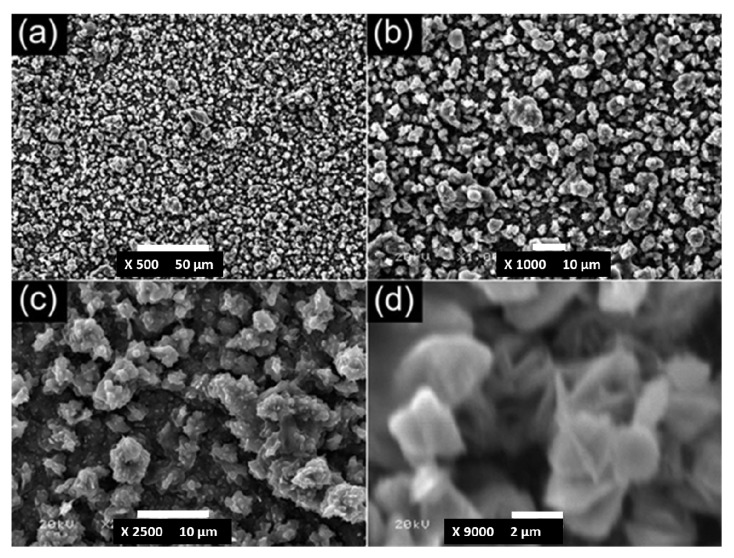
SEM images with various magnifications of the as-prepared copper surface after surface oxidation. Images reprinted from [112], with permission from AIP Publishing LLC, Copyright 2008.

**Figure 13:**
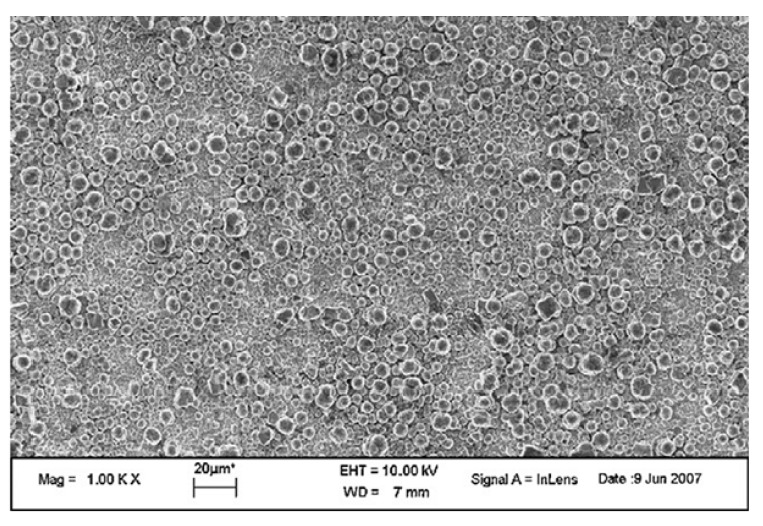
SEM image of the copper surface electroplated at the current density of 0.08 A/cm^2^. Images reprinted from [114], with permission from Elsevier, Copyright 2009.

**Figure 14:**
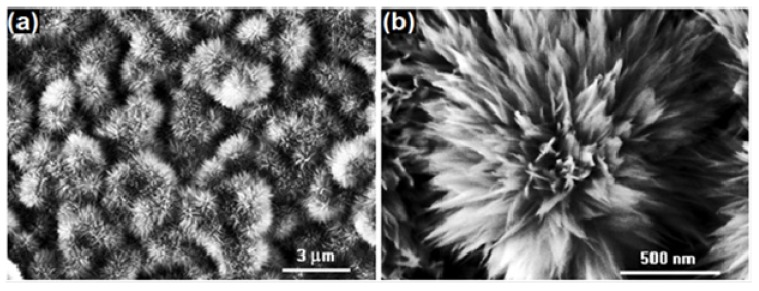
(**a**) Low and (**b**) high magnification SEM images of the boehmite film on the aluminum foil prepared using the solution-phase synthesis method. Images reprinted from [117], with permission from Elsevier, Copyright 2011.

A surface oxidation of metals by various acid and base treatments is one of the most simple and cost-effective ways to develop hierarchical superhydrophobic surfaces. Guo’s research group performed simple surface oxidation of copper after immersing in a 60 mL aqueous solution of potassium persulfate (0.065 M) and potassium hydroxide (2.5 M) at 60 °C for about 10–30 min. which resulted into the growth of hierarchically structured CuO microcrystals and the surface chemical modification by vinyl terminated poly(dimethylsiloxane) (PDMSVT) changed the wettability of the copper substrate from hydrophilic to highly superhydrophobic [112]. Hierarchical structures having microflowerlike protrusions with a diameter in the range of 2–8 µm are formed uniformly after surface oxidation of the copper which is similar to that of a lotus leaf (Figure 12). This surface showed stable superhydrophobicity in strong acid and base environments. Guo’s research group [113] also reported relatively similar lotus leaf-like surface morphology created on aluminum and aluminum alloys by treating both by NaOH (1 M) for 2 h and reducing the surface energy by perfluorononane (C_9_F_20_) and PDMSVT, respectively. Xi and co-workers [114] reported superhydrophobic lotus leaf-like microstructured surfaces on hydrophilic copper substrates using electroplating in aqueous solution composed of 0.08 M CuSO_4_ and 0.06 M H_2_SO_4_ without any further surface chemical treatment. At the applied current density of around 0.08 A/cm^2^, quasi-spherical grains having the diameters of 5–10 µm were observed distributed on the surface with mean central distances between these grains of about 20 µm (Figure 13), which is similar to the papillae diameter and mean central distance between papillae observed on the lotus leaf. This electroplated copper surface resembling the surface microstructure of the lotus leaf showed superhydrophobic behavior with a water contact angle of 153.58° and a sliding angle of 7.98° without any surface chemical modification. The authors suggested that the surface structure configuration should be fabricated suitably to achieve superhydrophobicity on hydrophilic surfaces without surface chemical modification (Marmur’s hypothesis) [115]. Wu and Shi [116] have reported the lotus leaf-like micro-nanoscale binary surface morphology of copper phosphate dihydrate films prepared by galvanic cell corrosion of a copper foil with aqueous phosphoric acid (0.05 M) drops. The as-prepared film showed a bicontinuous porous network consisting of 80 nm thick dendritic crystal nanosheets. These nanosheets are aligned with their planes perpendicular to the substrate. This surface showed strong superhydrophilic wettability which was transformed to superhydrophobic by simply heating or modifying the surface with monolayers of *n*-dodecanethiol. Liu *et al.* [117] have prepared hierarchical boehmite films on aluminum foil by a simple solution-phase synthesis method. The aluminum foil was immersed in the autoclave containing aqueous solution of AlCl_3_ (50 mM) and triethanolamine (0.75 M) and heated in the oven at 100 °C for 5 h. The prepared film showed three dimensional flower-like microprotrusions (diameters ranging from 1 to 3 µm) assembled from several nanoneedles (Figure 14), which is identical to the microstructure of lotus leaves. The surface acquires a dual scale hierarchy, one from micrometer scale protrusions and other from the dimensions of the nanoneedles on the order of nanometers. Although this surface initially exhibited superhydrophilic behavior, it converted into a superhydrophobic state (water contact angle of 169° and a sliding angle of 4°) after stearic acid modification. This film also showed long-term storage stability and better mechanical durability.

**Figure 15:**
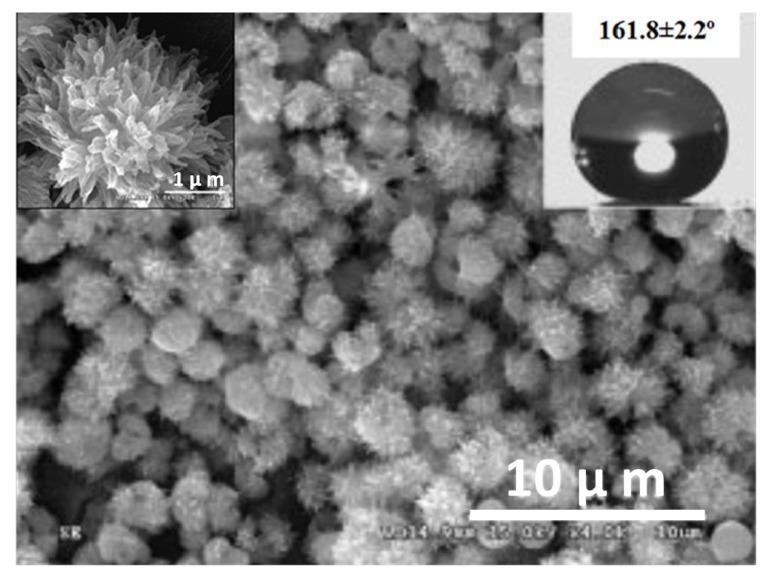
SEM image of coatings cast at room temperature with RH of 75%, left inset shows enlarged view of a single micro-flower on coating and right inset shows the water contact angle on this surface. Images reprinted from [118], with permission from WILEY-VCH Verlag GmbH & Co. KGaA, Weinheim, Copyright 2005.

**Figure 16:**
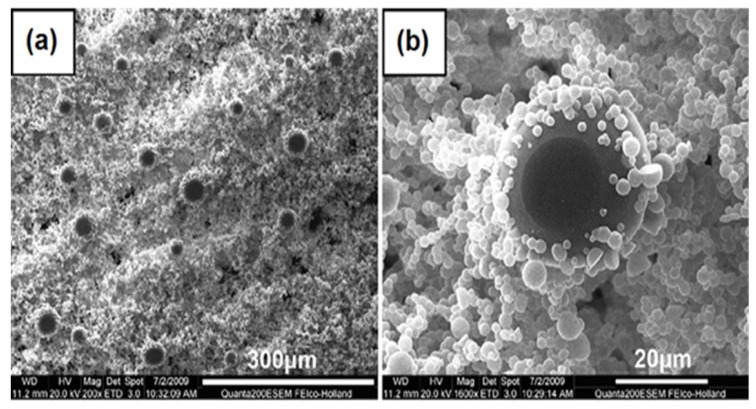
(**a**) Low and (**b**) high magnification SEM images of the copolymer film prepared from 50% (*v/v*) ethanol content in copolymer solution. Images reprinted from [119], with permission from Elsevier, Copyright 2010.

Zhao *et al.* [118] reported simple one step fabrication method for superhydrophobic coatings by casting bisphenol A polycarbonate (PC) solution under a humid atmosphere (RH~75%). A vapor-induced phase separation resulted during the solidification and a lotus leaf-like hierarchical micro-nano-binary structure (MNBS) was developed. The coating was developed by the random stacking of the flower-like particles on the surface having diameter in the range of 2–6 µm (Figure 15). This microstructure is identical to that of the papillae structure present on the natural lotus leaf. An excellent superhydrophobicity with a water contact angle of 161.8 ± 2.28° and a sliding angle of 9.4 ± 2.68° was achieved without subsequent chemical modification. Wei and co-workers [119] synthesized styrene and 2,2,3,4,4,4-hexafluorobutyl methacrylate copolymers by bulk polymerization and the superhydrophobic copolymer films were prepared using a phase separation method. The surface roughness of the films was controlled by the degree of phase separation using ethanol. The film prepared from 50% (*v/v*) ethanol content in the copolymer solution showed rough surface morphology similar to that of a lotus leaf (Figure 16), exhibiting excellent superhydrophobicity with a high water contact angle (154.38°) and a low sliding angle (5.88°).

**Figure 17:**
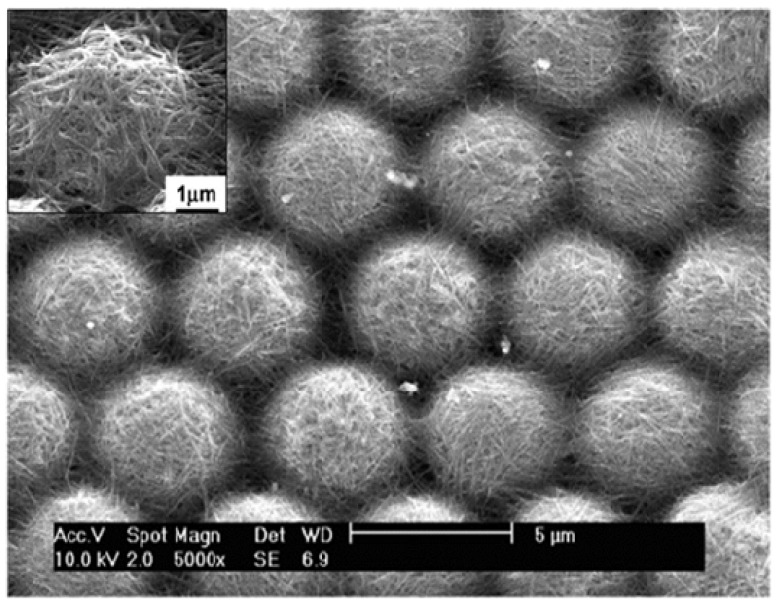
Surface morphology of the bionic surface prepared with PS microsphere/SWCNTs composition arrays, inset shows the higher magnification image of SWCNTs decorated microsphere. Images reprinted from [120], with permission from American Chemical Society, Copyright 2007.

Li and co-workers [120] reported for the first time the development of hierarchical bionic (lotus leaf-like) surface from the combination of polystyrene (PS) and carbon nanotubes (CNTs), where the CNTs with a concentration of 2.0 mg/L were decorated on monolayer PS colloidal crystal with periodicity of 5.0 µm by a wet chemical self-assembly method. Dense single walled CNTs (SWCNTs) were adsorbed like an interlaced ‘net’ structure on the hexagonally close packed PS microspheres and the sphere joints (Figure 17). This prepared hierarchical surface showed strong superhydrophobic behavior after surface chemical modification using 1*H*,1*H*,2*H*,2*H*-perfluorodecyltrichlorosilane. The well distributed CNTs on PS spheres have unique electrical and electrochemical properties having large specific area and can be used as gas sensors with good selectivity and great sensitivity.

**Figure 18:**
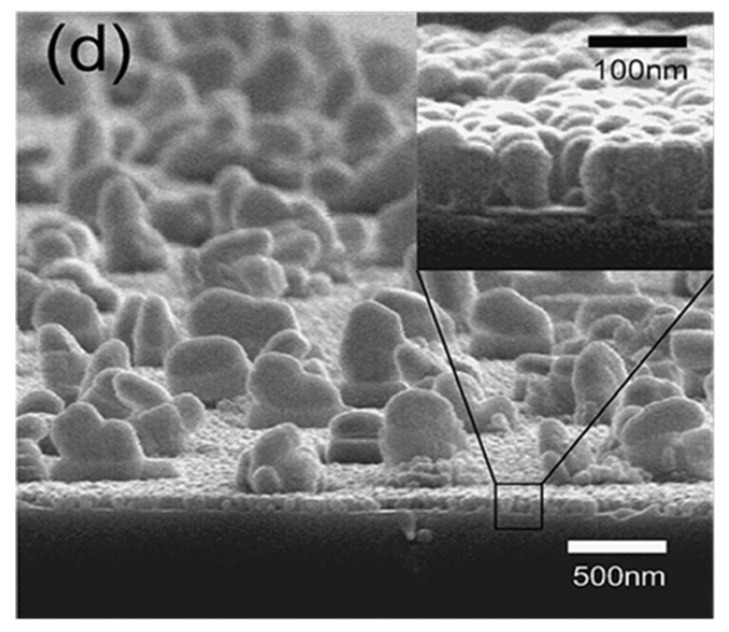
SEM image of Si wafer surface etched at 150 W r.f. power using CF_4_ gas at 5 Pa with the Cu nanodots. Images reprinted from [122], with permission from Elsevier, Copyright 2007.

Liu *et al.* [121] used a novel emulsion-mediated sol–gel process to synthesize micro-nanoscale binary structured composite particles of silica/fluoropolymer. These composite particles can be applied on different substrates using spin or spray coating techniques to mimic the lotus leaf-like hierarchical micro-nanostructure. These surfaces showed excellent non-wettability against water with a water contact angle larger than 150°. Kim and co-workers [122] developed superhydrophobic surfaces exhibiting very low contact angle hysteresis (<5°) by combining nano-scale surface roughening with a hydrophobic a-C:H:Si:O coating. A thin Cu film was deposited on a Si (110) wafer by DC magnetron sputtering and this Cu film was converted into nano-sized dots by rapid thermal annealing (at 550 °C for 15 min) in a pure hydrogen environment which acts as mask during CF_4_ plasma etching of Si. After CF_4_ plasma etching at 5 Pa with Cu nano-dots as the mask, lotus leaf-like rough structures were obtained as shown in Figure 18, where coarse posts are formed on the nano-posts. A hydrophobic a-C:H:Si:O coating on this surface structure has revealed superhydrophobic wetting behaviour.

## 5. Conclusions and Future Perspectives

Wonderful things can result from continuously learning from Nature. The discovery of the principle behind the extreme superhydrophobicity of lotus leaves by Barthlott and Neinhuis at the end of 20th century added fuel to the then slow-burning research on superhydrophobicity. The superhydrophobicity of the lotus leaf is mainly due to the low surface energy provided by epicuticular wax crystalloids and the air pockets trapped in micrometer-scale papillae structure which minimizes the solid-water contact area. Research on the development of superhydrophobic surfaces exhibiting similar wetting properties to lotus leaves is a critical and promising subject in materials science. In this review article, we tried to cover the different wetting properties of lotus leaves and the abundant research work carried out (since 1997) on the development of superhydrophobic surfaces by mimicking the lotus leaf-like surface morphology. So far, perfect mimicry of the lotus leaf surface morphology has not yet observed/reported. The mimicry of lotus leaf micro/nanostructure is just limited to the achievement of high water contact angles and low sliding angles on the surface, however, serious steps are to be taken to achieve a durability similar to that of a lotus leaf. Most of the superhydrophobic surfaces developed by mimicking lotus leaf-like micro-nanostructure have used various polymers during synthesis due to their natural hydrophobicity and toughness. It has been well demonstrated that plenty of naturally occurring surfaces are superhydrophobic and they all are developed with polymers [123,124]. The templation method is promising to achieve near-perfect mimicry of the lotus leaf-like morphology, whereas other physical and/or chemical methods could partially mimic the surface structure. The mimicry was either near-perfect or partial, however the wetting properties were identical to those of the lotus leaf.

A main challenge in the development of the superhydrophobic surfaces is the design of their surface micro/nanostructures and low surface energy. Off course, the lotus leaf-like surface morphology is ideal to achieve strong superhydrophobicity on solid surfaces but further research on many other aspects should be carried out in order to compete in the commercial market. Enormous amounts of research on lotus leaf-like superhydrophobic coatings have been successfully carried out on the small scale in the laboratory, however they can be ineffective when applied on the large scale due to several reasons. The mechanical durability or scratch resistance of the coatings in combination with optical transparency is needed for applications like self-cleaning coatings on windows and door glasses of buildings. The adherent coating is paramount in the application of anticorrosive coating on the metals. We believe that, instead of inorganic superhydrophobic coatings, the future superhydrophobic research should be directed towards polymeric superhydrophobic coatings which shows good adhesion with underlying substrates of any shape and size and can last longer with high mechanical durability and optical transparency. The demand for superhydrophobic coatings is growing, and this will perhaps be one of the most challenging and interesting research fields in materials science for the next two decades.
